# METTL3 as a potential therapeutic target in gastric cancer

**DOI:** 10.3389/fonc.2024.1483435

**Published:** 2024-11-29

**Authors:** Zhefei Yu, Yang Yang

**Affiliations:** The First Affiliated Hospital of Guangxi University Of Chinese Medicine, Nanning, Guangxi, China

**Keywords:** METTL3, m6A, gastric cancer, angiogenesis, glycolysis, tumour microenvironment, drug resistance

## Abstract

Gastric cancer (GC) is one of the leading causes of cancer-related death worldwide. N6-methyladenosine (m6A) modification is the most prominent epigenetic modification of eukaryotic mRNAs, and methyltransferase-like 3 (METTL3), a core component of the methyltransferase complex, catalyzes m6A modification. The results of previous studies indicate that the expression level of METTL3 is significantly elevated in gastric cancer tissues and cells. In addition, fluctuations in m6A levels induced by METTL3 are closely associated with the malignant progression of tumors as well as the poor prognosis of patients with gastric cancer. In this review, we focus on the potential mechanism of METTL3 in gastric cancer, and through our analysis, we suggest that targeting METTL3 could be a new therapeutic tool for treating GC.

## Introduction

Gastric cancer (GC) is one of the top five malignant tumors worldwide and is very likely to cause death in cancer patients (1). The cause of stomach cancer varies by country. Asia, especially Japan and Mongolia, has the highest age-standardized rates of incidence and mortality, followed by Central and Eastern Europe and South America (2). In recent years, the incidence of GC in adults aged < 50 years has been gradually increasing in both low-risk and high-risk countries, with an estimated > 1 million new cases per year (3). Poor lifestyle habits and medical history, such as Hp infection, smoking status, smoking history, alcohol consumption status, alcohol consumption history, and pickled food intake, may contribute to the development of GC (4). N6-methyladenosine (m6A) is a form of mRNA modification that works by methylating the sixth nitrogen atom of RNA adenosine, thereby regulating RNA metabolism, which mostly occurs in eukaryotes. The methyltransferase complex is responsible for catalyzing m6A modification and relies on a subunit called methyltransferase-like 3 (METTL3) The core component of the methyltransferase complex is composed of METTL3, METTL4, and WTAP. METTL3 and METTL14 form a heterodimer in which WTAP plays a regulatory role. METTL3 has catalytic activity, and adenosylmethionine (SAM) binds to the catalytic domain of METTL3 through a gate loop to provide methyl. METTL14 provides structural support near the active site to help maintain the stability and integrity of the complex. METTL3 and METTL14 jointly recognize the substrate RNA (5). m6A is composed of 580 amino acids and contains two domains that are important for enzymatic activity: the zinc finger domain and the methyltransferase domain (6). METTL3 has been reported to be important in promoting tumorigenesis, accelerating the proliferation and metastasis of tumor cells, and promoting stem cell differentiation in a variety of cancers (7). METTL3 regulates RNA processing, splicing, metabolism, and ribonucleoprotein complex biogenesis through m6A modification (8). m6A in the nucleus promotes the maturation of pre-mRNA by regulating the splicing process. The transcribed mRNA is exported to the cytoplasm for translation under the action of m6A. m6A in the cytoplasm promotes the translation of mRNA in many ways, and can also maintain mRNA stability or promote mRNA degradation (9). Several studies have shown that abnormal changes in genes involved in RNA methylation may induce gastric cancer in healthy individuals or have adverse consequences in patients with gastric cancer (10). METTL3 promotes drug resistance and proliferation, invasion, migration, and metastasis in GC cells; inhibits apoptosis; and affects the expression of key components of various signaling pathways that regulate GC cell proliferation. In addition, METTL3 can be used as a reliable marker for clinicians to evaluate disease development trends in patients, and targeting METTL3 has gradually become a treatment modality for GC patients (11–13). Therefore, we summarize the research progress on METTL3 in GC tumorigenesis and propose that interfering with METTL3 expression may be a novel approach for treating patients with GC.

## Results

### METTL3 inhibits downstream gene expression in gastric cancer

BATF2 contains a structural domain called the basic Leu zipper protein and is encoded by a gene located on human chromosome 11q (14). BATF2 blocks tumor progression through multiple mechanisms, such as the inhibition of epithelial–mesenchymal transition (EMT), CCN1 transcription, c-Jun expression, and angiogenesis (15–17). By catalyzing m6A modification of the MUT3 site in the 3’ untranslated region (3’UTR) of BATF2, METTL3 maintains BATF2 mRNA stability and inhibits BATF2 expression. BATF2 has been shown to inhibit GC cell growth, metastasis, and proliferation by prolonging the cell cycle both *in vivo* and *in vitro*. Reduced BATF2 expression is usually associated with poor prognosis in patients with GC. Human phospho-kinase microarray assays revealed that BATF2 overexpression decreased ERK phosphorylation (Thr202/Tyr204); conversely, BATF2 knockdown increased ERK phosphorylation. Western blotting (WB) and immunohistochemistry confirmed that high p-ERK levels were detected in patients with low BATF2 expression. WB demonstrated that the cell cycle proteins D1 (cyclin D1), MMP2, and MMP9 are targets of ERK signaling. These proteins and p-ERK were decreased and increased in BATF2-overexpressing and BATF2-knockdown cells, respectively. Overall survival of GC patients in an internal cohort classified according to BATF2 and p-ERK expression revealed that the level of p-ERK determines the prognostic value of BATF2. BATF2 regulates p53 protein levels via posttranslational modifications. BATF2 deletion promotes ubiquitylation-mediated p53 degradation, which can activate ERK signaling and promote ERK phosphorylation and the expression of MMP2, MMP9, and cyclin D1, accelerating GC cell proliferation, invasion, and metastasis (18).

RNA processing includes transcription initiation, splicing, and termination. Pre-mRNAs are spliced to generate mature mRNAs in one of the most important steps in eukaryotic RNA processing, and the splicing process is controlled by splicing factors. METTL3 downregulates the expression of splicing factors and is overexpressed in cancer tissue. Reduced SRSF11 and TRA2A expression is positively correlated with poor survival in patients with stomach adenocarcinoma. GSEA demonstrated that downregulation of the splicing factor RSF11 affects oncogenesis by regulating the expression of genes associated with the p53/apoptosis, inflammation/immune response, and UV/ROS stimulus response pathways. According to the m6A target database and eCLIP-seq data, several m6A-reading proteins, including IGF2BP1/2/3, YTHDC1/2, and HNRNPC, interact with SRSF11 mRNA. At the molecular level, the specific mechanism of SRSF11 expression related to the regulatory role of METTL3 needs to be elucidated in further studies (19). As a downstream effector of METTL3, ADAMTS9 is regulated by METTL3. METTL3 inhibits ADAMTS9 expression via YTHDF2-dependent mRNA degradation. When this occurs, the PI3K/AKT pathway is noticeably inactivated, and ADAMTS9-mediated downregulation of AKT and PI3K phosphorylation is attenuated, thereby promoting GC progression (20). METTL3 targets ANGPTL3 and downregulates its expression, which is conducive to tumor growth, proliferation, migration, and invasion (21) (**Table 1**). In this study, we noted that genes downstream of METTL3 are closely related to the stability of p53. The specific mechanism by which these genes regulate p53 is still unclear and needs to be further explored.

**Table 1:** Overview of related genes negatively regulated by METTL3.

Downstream genes of METTL3	Inhibit/promote	Reader	Mechanisms	Role of METTL3	Role of the downstream genes	Reference
BATF2	inhibit	/	Promotes ubiquitination-mediated p53 degradation and P-ERK	oncogene	tumour suppressor	(18)
SRSF11	inhibit	IGF2BP1/2/3, YTHDC1/2, HNRNPC	Regulation of gene expression associated with p53/apoptosis, inflammatory/immune responses, and UV/ROS-stimulated response pathways	oncogene	splicing factor	(19)
ADAMTS9	inhibit	YTHDF2	Inactivates the PI3K/AKT signaling pathway	oncogene	tumour suppressor	(20)
ADAMTS9	inhibit	YTHDF2	Promotes angiogenesis	oncogene	tumour suppressor	(20)
RIG-I signaling pathway-related genes	inhibit	IGF2BP2	METTL3 inhibits the RIG-I-like signaling pathway, attenuates T-cell cytotoxicity, and weakens its ability to kill cancer cells	oncogene	tumour suppressor	(37)

**Table 1** METTL3, methyltransferase-like 3.

### METTL3 promotes the expression of downstream genes

MeRIP-seq, RNA-seq, and TCGA analyses revealed that PBX1 is a downstream target of METTL3 methylation. METTL3-mediated m6A modification of the 5’UTR and 3’UTR of PBX1 mRNA promotes the expression of PBX1 mRNA by maintaining its stability, subsequently promoting the malignant progression of GC. GCH1 is among the genes with higher expression levels in GC tissues than in normal tissues, and GCH1 is the rate-limiting enzyme for BH4 biosynthesis. High PBX1 antibody enrichment was detected in the GCH1 promoter region, and METTL3-overexpressing GC cells with high GCH1 expression also presented high GCH1 expression. However, the expression of GCH1 decreased significantly as a result of PBX1 knockdown. In conclusion, GCH1 functions downstream of the METTL3-PBX1 axis. PBX1 binds to a site 500 bp away from the transcription start site of GCH1 and induces GCH1 synthesis. The GCH1 protein regulates BH4 biosynthesis in GC cells, leading to the proliferation, invasion, migration, and metastasis of GC. BH4 can regulate cellular metabolism and has antioxidant effects, which is why it can progress to gastric cancer, but its specific function remains to be explored (22). The mechanism by which METTL3 induces an increase in BH4 levels is well understood. However, further research is needed to explore the specific mechanism by which BH4 affects gastric cancer cells.

METTL3-mediated m6A methylation occurs at the stop codon of ZMYM1 mRNA and enhances ZMYM1 expression via a m6A-HuR-dependent pathway. ZMYM1 binds to E-cadherin by recruiting the CRS site of the CtBP/LSD1/CoREST complex to the promoter, thereby inhibiting E-cadherin expression. It also promotes EMT and metastasis in GC cells (23). The results of existing studies suggest that the expression level of E-cadherin decreases when the EMT program is initiated. At present, it is only inferred from this conclusion that the decrease in E-cadherin expression indicates that the EMT program is initiated, but the mechanism that causes the change in EMT marker levels is still unclear. Further research is needed to confirm this hypothesis.

SPHK is a protein-regulated cellular metabolic process in which phosphorylated sphingosine catalyzes the synthesis of sphingosine 1-phosphate. SPHK2 is associated with tumorigenesis, cancer progression, and chemoresistance. KLFs are TFs with Cys-2/His-2 zinc-finger-like structures that bind to the CACC or GT box. Abnormal expression of KLF2 has been detected in many tumors and has an important impact on tumorigenesis and development. METTL3 promotes m6A modification of SPHK2 target genes, and YTHDF1 binds to m6A-containing SPHK2 mRNA to form a translation initiation complex by interacting with eukaryotic initiation factor 3a (elF3a) to promote the translation of SPHK2 mRNA. These results suggest that the translation of SPHK2 is promoted by METTL3 and YTHDF1. SPHK2 promotes the ubiquitination of KLF2, which is facilitated by SPHK2-mediated KLF2 phosphorylation. Therefore, SPHK2 induced KLF2 degradation. K122 is the main ubiquitinated residue of KLF2, and Thr173 is the main phosphorylated residue of KLF2. Therefore, SPHK2 promotes the migration, proliferation, and invasion of GC cells (24). METTL3 binds to the 3’UTR of DEK to induce m6A modifications. METTL3 stabilizes DEK mRNAs to promote DEK expression. Thus, METTL3 promotes GC cell proliferation, migration, and metastasis (25). HBXIP can upregulate the expression of METTL3 and promote MYC expression (26). MYC is a downstream target of METTL3. MCM5 and MCM6 are key components of MYC. They assemble into complexes that initiate eukaryotic genome replication. High expression of MCM5 is correlated with poor clinicopathological parameters and survival METTL3 may regulate MYC through directly. For instance, promoting mRNA expression by maintaining the stability of mRNA. Besides, METTL3 may also indirectly regulate MYC. Therefore, the relationship between METTL3 and MYC in gastric cancer is still unclear and still needs further exploration (27). METTL3 modifies YAP1 to promote GC proliferation and metastasis. YAP1 is involved in the YAP signaling pathway through m6A. A new direction and target for GC treatment was developed with the discovery of the METTL3–YAP1 pathway (28) (**Table 2**). The mechanism by which METTL3 regulates DEK and SPHK2 expression has been elucidated. METTL3 maintains the stability of DEK mRNAs and enriches m6A-modified SPHK2 mRNAs by binding to YTHDF1 and elF3a. The transcript stability of MYC-associated genes decreased because of the depletion of METTL3. This conclusion is speculative, and the mechanism of action of METTL3 and MCM5/MCM6 should be investigated in the future.

**Table 2:** Overview of related genes positively regulated by METTL3.

Downstream genes of METTL3	Inhibit/promote	Reader	Mechanisms	Role of METTL3	Role of the downstream genes	Reference
PBX1	promote	/	Promotes GCH1 expression and induces BH4 synthesis	oncogene	transcription factor	(22)
ZMYM1	promote	HuR	Recruitment of the CtBP/LSD1/CoREST complex inhibits E-cadherin expression and promotes EMT program initiation	oncogene	DNA binding protein	(23)
SPHK2	promote	YTHDF1、elF3a	Inhibit the expression of KLF2	oncogene	oncogene	(24)
DEK	promote	/	Promote the expression of DEK	oncogene	oncogene	(25)
MYC	promote	/	Still need to be explored	oncogene	oncogene	(26)
YAP1	promote	/	Activation of YAP signaling pathway	oncogene	oncogene	(28)
NDUFA4	promote	IGF2BP1	Promotes glycolysis, regulates mitochondrial dynamics and biogenesis, inhibits apoptosis	oncogene	oncogene	(35)
HDGF	promote	IGF2BP3	Transcriptional activation of glucose metabolism-related genes GLUT4 and ENO2 promotes glycolysis	oncogene	oncogene	(30)
SUV39H2	promote	IGF2BP2	Increased DNA damage repair leading to resistance to cisplatin	oncogene	H3K9 selective histone methyltransferase	(40)
PARP1	promote	YTHDF1	Increased DNA damage repair leading to resistance to oxaliplatin	oncogene	Organizational core functional genes	(41)

**Table 2** elF3a, eukaryotic initiation factor 3a; EMT, epithelial–mesenchymal transition; METTL3, methyltransferase-like 3.

### Upstream regulators of METTL3

**Figure 1:**
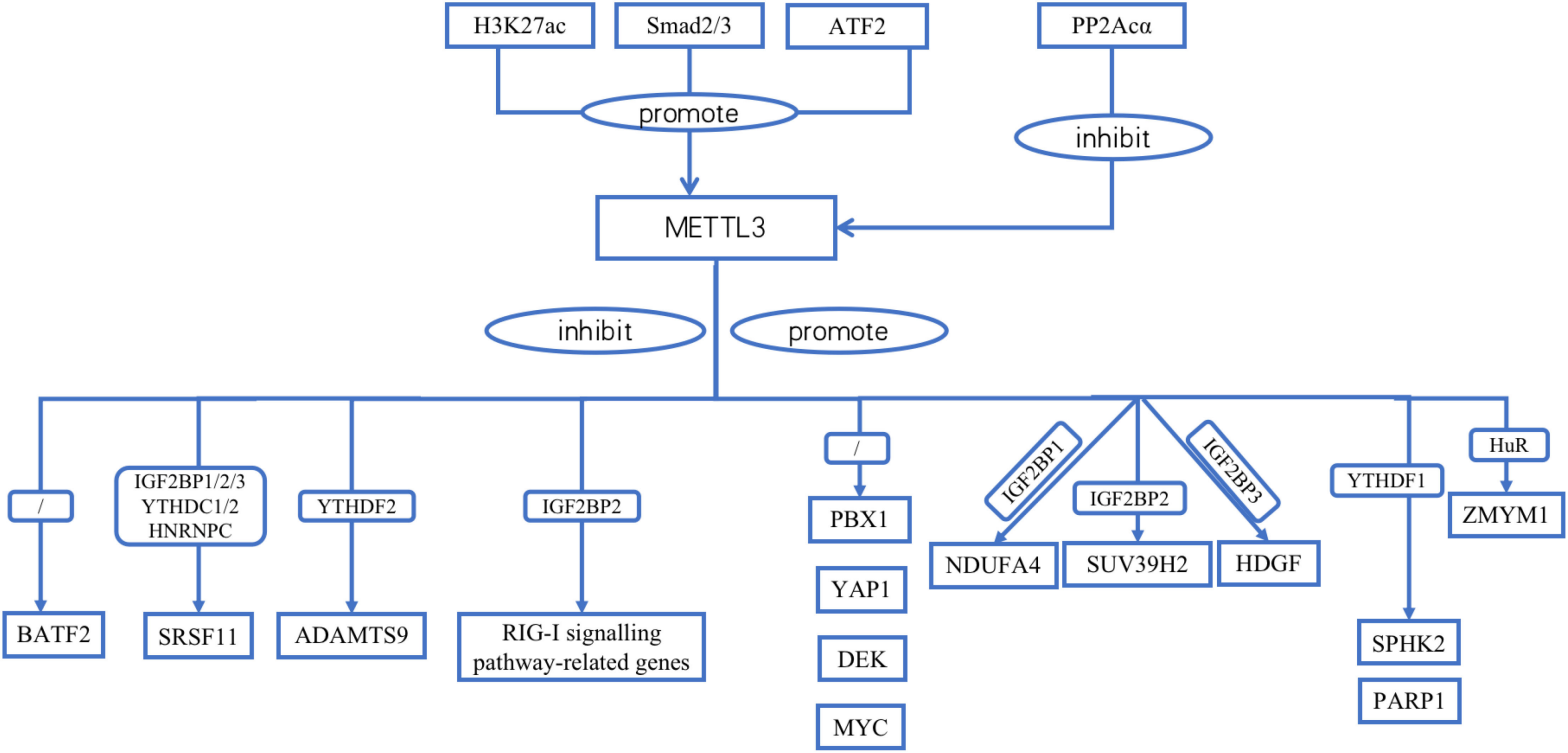
Relationships between METTL3 and upstream/downstream molecules.

METTL3 contains many upstream regulators. Homologous box (HOX) genes are involved in cell proliferation, migration, metabolism, and apoptosis. HOXA10 binds to the promoter, increases the activity of the TGF-β2 promoter and activates the expression of TGF-β2 through transcriptional regulation. This, in turn, increases the secretion of TGF-β2 in the extracellular space, activates TGF-β/Smad2/3 signaling, and leads to a significant increase in Smad2/3 expression in the nucleus. Smad2/3 bind directly to the METTL3 promoter region in the nucleus. Smad2/3 is involved in the transcriptional regulation of METTL3, promotes METTL3 expression, and promotes m6A RNA modification in GC cells (29). H3K27 acetylation in GC cells is catalyzed by P300. P300 binds to acetylated H3K27, together with the METTL3 promoter region, positively regulating METTL3 expression (30). PP2Acα can remove the phosphoryl group added to the METTL3 serine 43 site (S43) via ataxia telangiectasia mutated (ATM) kinase and inhibits p-ATM. Moreover, PP2Acα can increase the activity of ATM kinases, resulting in the downregulation of METTL3 (31). The transcription factor ATF2 promotes the transcription of METTL3 by binding to the METTL3 promoter region. WB revealed that ATF2 overexpression increased cyclin D1 expression. However, METTL3 knockdown dampened this effect. The downregulation of ATF2 led to a decrease in cyclin D1 expression. This effect was counteracted by the upregulation of METTL3. Therefore, METTL3 is associated with cell cycle progression. Moreover, METTL3 activated cyclin D1 expression. ATF2 activates the METTL3/cyclin D1 signaling pathway to promote GC cell proliferation and inhibit GC cell apoptosis (32). PLAGL2 attaches to the upstream promoter of UCA1 to aid its transcription. UCA1 mRNA may function as a microRNA (miRNA) sponge that adsorbs miR-145-5p bound to YTHDF1 mRNA, upregulates YTHDF1 expression, promotes snail expression through METTL3 methylation, activates EMT, and promotes GC metastasis (33) (**Table 3**; **Figure 1**).

**Table 3:** Overview of related genes that regulate METTL3.

Upstream genes of METTL3	Inhibit/promote	Mechanisms	Reference
Smad2/3	promote	Binds to the promoter of METTL3 and promotes its transcription	(29)
H3K27ac	promote	Binds to the promoter of METTL3 and promotes its transcription	(30)
PP2Acα	inhibit	Remove the phosphate groups that are added to METTL3 serine or threonine in the presence of ATM kinase, and downregulates METTL3 levels	(31)
ATF2	promote	Binds to the promoter of METTL3 and promotes its transcription	(32)

**Table 3** ATM, ataxia telangiectasia mutated; METTL3, methyltransferase-like 3.

### Effects of METTL3 on angiogenesis in gastric cancer

Angiogenesis is an important factor in tumor formation, rapid multiplication, and cancer progression. Vascular endothelial growth factor is at the forefront of this process. Vascular endothelial growth factor is secreted by tumor cells and lymphocytes and is an important component of tumor angiogenesis (34).

ADAMTS9 has been shown to be a downstream effector of METTL3. The degradation of ADAMTS9 is usually due to the action of the YTHDF2-dependent pathway. METTL3 represses ADAMTS9 expression by degrading YTHDF2-dependent mRNAs, limiting the ability of ADAMTS9 to promote GC progression via the PI3K/AKT pathway. ADAMTS9 can induce notable inactivation of the PI3K/AKT signaling pathway in gastric cancer cells by inhibiting its phosphorylation (20). The results of this study suggest that METTL3 acts as an oncogene to promote angiogenesis by targeting gastric cancer-related enzymes.

### Effects of METTL3 on glycolysis in gastric cancer

Cell survival and other functional activities are energy-intensive processes. Glycolysis and oxidative metabolism release large amounts of energy for cellular use. Aerobic glycolysis, which involves the oxidative phosphorylation of mitochondria, affects the growth of tumor cells. METTL3 promotes m6A methylation of NDUFA4 through a reader called IGF2BP1. This mechanism allows IGF2BP1 to attach to the 3’-UTR of NDUFA4 and then promote NDUFA4 expression. In GC cells, NDUFA4 may promote glycolysis and oxidative metabolism. NDUFA4 can increase oxygen consumption and extracellular acidification rates, which are important indicators of glycolysis for assessing the metabolic state of the cell and energy metabolism. ENO1 and LDHA, two enzymes involved in glycolysis, also increase the expression of NDUFA4 and improve glycolysis and oxidative metabolism in GCs.

The ratio of the mitochondrial membrane potential (MMP) to reactive oxygen species (ROS) is an important signal for normal physiological functions of the cell and for cellular damage caused by environmental factors. Once the ROS level exceeds the capacity of endogenous antioxidant defenses, redox homeostasis is disrupted, which leads to structural or conformational changes in DNA, lipids, and proteins, ultimately leading to cell death. OPA1, p-Drp1, and PGC1α are associated with mitochondrial fusion, fission, and biogenesis. NDUFA4 overexpression increases the MMP; decreases ROS levels; increases OPA1, p-Drp1, and PGC1α levels; promotes mitochondrial fusion, fission, and biogenesis; and inhibits apoptosis by regulating mitochondrial dynamics and biogenesis in gastric cancer cells (35). Wang et al. reported that in METTL3-overexpressing BGC823 cells, the expression of the proliferation biomarker Ki-67 and the angiogenesis marker CD31 was significantly increased. Moreover, angiogenesis, human umbilical vein endothelial cell growth, and tube formation were significantly promoted by METTL3 overexpression. In experiments with tumor xenografts of the HGC-27 and NCI-N87 cell lines, METTL3 overexpression promoted increases in tumor volume and weight *in vivo*. In summary, METTL3 acts as an oncogene that helps GC cells generate blood vessels, proliferate, and metastasize. Its function is dependent on m6A catalytic activity (the catalytic mutant of METTL3 does not have any regulatory effects). Analysis of TCGA data revealed that H3K27 acetylation in GC cells is catalyzed by P300. The METTL3 promoter region is rich in P300 binding and H3K27ac signaling, and P300 positively regulates METTL3 transcription and expression. In summary, P300 binds to the promoter region of METTL3 and acetylates H3K27 to positively regulate METTL3 expression. HDGF was identified as a downstream target of METTL3, and its expression was increased by m6A modification. This process is mediated by METTL3 and is inseparable from the involvement of IGF2BP3. When an HDGF overexpression plasmid was transfected into METTL3 knockout cells via plasmid transfection, glycolytic activity was significantly increased (30). Additionally, a greater amount of lactic acid was produced. Mechanistically, HDGF binds to the promoter regions of genes related to glucose metabolism, including GLUT4 and ENO2. HDGF transcription subsequently activates GLUT4 and ENO2 expression, inducing glycolysis in GC cells. This ultimately facilitates GC progression and hepatic metastasis (30). Cellular energy metabolism requires the participation of proteins. Gene ontology enrichment revealed that these proteins were composed mainly of proteins whose expression was reduced under the influence of METTL3 overexpression. These proteins trigger glycolysis in GC cells and promote tumor progression by mediating mitochondria-associated oxidative phosphorylation (36). METTL3 addresses the energy and biosynthetic needs of the ongoing proliferation and metastasis of malignant tumors by regulating glycolysis-related enzymes. Additionally, NDUFA4 inhibits apoptosis by promoting mitochondrial fission.

### Effects of METTL3 on the tumour microenvironment of gastric cancer

Exosomes are tiny vesicles released into the extracellular space by almost all cell types and contain DNA, mRNAs, proteins, lipids, miRNAs, and other bioactive substances that are unique to the original cell. These cargoes can build bridges for cells to communicate with each other and participate in various physiological and pathological processes. In the tumour microenvironment (TME), many interactions and communications occur between cells. Recently, increasing evidence has shown that xenobiotics play key roles in this process. THBS1, an angiogenesis inhibitor, is negatively associated with cancer progression. Cytokines are small-molecule proteins. When stimulated, immune and non-immune cells can synthesize and secrete cytokines. They are biologically active, can promote cell growth, and can regulate adaptive immunity. Cytokines can also be used to repair damaged tissues.

Interleukins, interferons, tumor necrosis factors, colony-stimulating factors, and growth factors are cytokines. Exosomes derived from GC cells are more likely to produce IFN-γ and TNF-α. THBS1 was found to stimulate cytotoxicity and cytokine production in Vγ9Vδ2 T cells. Thus, the toxicity of Vγ9Vδ2 T cells to cancer cells increases. Differentially expressed genes identified by RNA sequencing were associated with THBS1 expression. These genes are abundantly expressed in the RIG-I-like receptor signaling pathway. Similarly, in THBS1-OE-treated T cells, factors related to the RIG-I-like receptor signaling pathway were significantly increased in Vγ9Vδ2 T cells. Subsequent rescue experiments revealed that THBS1 regulated the function of Vγ9Vδ2 T cells, causing them to produce more cytokines. In addition, THBS1 can increase the toxicity of Vγ9Vδ2 T cells, thereby increasing their ability to kill GC cells. All the above is accomplished by THBS1 with the help of the RIG-I-like receptor signaling pathway. The SRAMP database was analyzed to confirm that m6A methylation occurs in genes involved in RIG-I signaling. THBS1 downregulated METTL3 in Vγ9Vδ2 T cells at the posttranscriptional level, resulting in increased expression of the reader IGF2BP2 and eraser FTO. The amount of METTL3 expressed in Vγ9Vδ2 T cells was approximately 4-fold greater than the amount of METTL3 expressed in FTOs. As a result, genes involved in the RIG-I signaling pathway are often methylated. THBS1 directly binds to METTL3 to reduce its expression level and the methylation of RIG-I signaling pathway genes. Genes related to the RIG-I signaling pathway are methylated by binding to the IGF2BP2 protein, which increases mRNA stability, promotes expression, activates the RIG-I signaling pathway, and regulates the function of Vγ9Vδ2 T cells. More cytokines subsequently increase the toxicity of Vγ9Vδ2 T cells, further promoting the synthesis and secretion of cytokines such as IFN-γ and TNF-α, thereby inhibiting cancer progression (37).

METTL3-mediated m6A modification and immunological infiltration are also strongly associated with the TME. The expression of PD-1 and PD-L1 is related to m6A regulator expression, according to Spearman correlation analysis. Using consensus clustering analysis, the GC samples were divided into two groups. These two groups of samples are involved in key signaling pathways, such as base excision, nucleotide excision and repair, and the cell cycle. Further experiments revealed that PD-L1 was more highly expressed in Cluster 1 than in Cluster 2. In addition, the infiltration of immune cells was greater in cluster 1. Thus, the number of m6A methylation regulators significantly affects the number of immune cells that infiltrate the TME. Genes that can affect the prognosis of patients with gastric cancer were first identified, and then a risk scoring model was established. This model was sufficiently convincing to predict the disease development status of patients with gastric cancer. WB demonstrated that METTL3 can increase the expression of PD-L1 in GC cells, but bioinformatics data suggest that the link between PD-L1 and METTL3 is weak; thus, m6A regulators may also be involved. The interactions between the TME and other variables need to be further explored (38). Different molecular subtypes of GC patients overexpress different core genes for METTL3 methylation, and the tumor mutational burden (TMB) and infiltration of immune cells are also not static. For patients with higher TMB, immunotherapy allows them to obtain greater therapeutic benefits and are more sensitive to the immune checkpoint inhibitor paclitaxel. Therefore, METTL3-mediated m6A methylation can influence the responsiveness of patients with GC to immunotherapy (39). Overall, METTL3 is a promising new therapeutic target for gastric cancer and may improve the efficacy of immunotherapy.

### Effects of METTL3 on the drug resistance of gastric cancer

DNA double-strand breaks are a type of DNA damage, and chemotherapeutic drugs exert their cytotoxic effects through this process. Cisplatin (CDDP) causes DNA double-strand breaks by forming platinum−DNA adducts, which is the mechanism by which CDDP induces cancer cell death. Yang et al. demonstrated that METTL3 increases the expression of SUV39H2 through methylation. SUV39H2 modifies the promoter region of DUSP6 by trimethylation of histone H3K9 to inhibit its transcription, reverse the dephosphorylation of DUSP6, activate homologous recombination repair, and enhance DNA double-strand break repair. In summary, SUV39H-induced DNA damage repair is the main cause of CDDP resistance. Targeting SUV39H2 can increase the sensitivity of tumor cells to CDDP and could become a new chemotherapy adjuvant (40). Cancer stem cells constitute one of the main causes of cancer recurrence. CD133+ tumor cells with high PARP1 expression presented oxaliplatin resistance. Studies have shown that METTL3 promotes stable PARP1 expression through m6A modification. PARP1 activates the base excision repair pathway to promote DNA damage repair, resulting in resistance of CD133+ gastric cancer stem cells to oxaliplatin. These findings provide a new approach for the clinical treatment of oxaliplatin-resistant patients with high METTL3 levels (41). XRCC1, PARP1, and RAD51 are important components of DNA repair. Wang et al. reported that these genes are positively correlated with METTL3 expression. Therefore, METTL3 promotes oxaliplatin resistance in gastric cancer cells via the DNA damage repair process (42). These findings suggest that chemotherapy resistance in gastric cancer is closely related to DNA double-strand break repair. These findings provide a new direction for the treatment of GC. In addition to SUV39H2 and PARP1, METTL3 is a promising new therapeutic target in GC.

### The relationship between METTL3 and non-coding RNAs in gastric cancer

miRNAs are upstream regulators of METTL3 expression. miR-1269b can target the METTL3 3’-UTR and negatively regulate the expression of METTL3 in GC cells (43). miR-4429 inhibits the expression of METTL3 by targeting METTL3 and reducing the methylation level of SEC62 mRNA, thus playing a role in inhibiting GC cell proliferation and enhancing GC cell apoptosis (44). miR-181-5p negatively regulates KLHL5 mRNA expression by binding to the 3’-UTR of KLHL5, reducing m6A levels and METTL3 expression (45). EED reversed the low expression of METTL3 by inhibiting the expression of miR-338-5p (46).

In addition to miRNAs, METTL3 contains several other upstream regulators. Circular RNAs (circRNAs) are important regulators of tumorigenesis that modulate the malignant behavior of tumor cells. Small noncoding RNAs encoded by Epstein–Barr virus (EBV) are found in Epstein–Barr virus (EBV)-associated GC (EBVaGC). Moreover, EBV also produces circular RNAs (ebv-circRNAs). The complex formed by circRPMS1 with Sam68 and P53 is enriched in the promoter of METTL3 and regulates the malignant behavior of EBVaGC cells by promoting the transcription of METTL3 (47, 48). The Epstein–Barr virus circRNA does not function as a miRNA sponge, and it directly interacts with the Sam68 protein. Therefore, Sam68 is expected to become a new target for the treatment of gastric cancer. However, P53 is a cofactor that interacts with Sam68, and the relationship between METTL3 and p53 still needs to be studied.

### The relationship between METTL3 and long non-coding RNAs in gastric cancer

Long noncoding RNAs (lncRNAs) are a class of noncoding RNAs that are more than 200 nucleotides long and do not have the ability to encode proteins. lncRNAs can bind to mRNAs, miRNAs, or proteins to regulate chromatin modification, transcription, and posttranscriptional and posttranslational regulation (49). lncRNAs have been shown to play critical roles in disease, viral infections, cell cycle differentiation, and metabolic processes (50).

lncRNAs are genes downstream of METTL3. METTL3 maintains the stability of THAP7-AS1 through posttranscriptional modifications (51). IGF2BP1 directly binds to and recognizes m6A modifications in METTL3-mediated ABLs and maintains their stability. ABLs in GC tissues promote gastric cancer cell survival and inhibit the apoptosis induced by various drugs (52). In GC induced by exposure to environmental chemical carcinogens, METTL3 promotes the expression of SNHG7 by increasing the m6A methylation level of the lncRNA SNHG7 and promotes the malignant progression of gastric cancer (53).

lncRNAs play a key role in the recruitment of METTL3. LINC02253 stabilizes KRT18 mRNA and upregulates KRT18 expression by promoting the recruitment of METTL3 to KRT18 mRNA, activating the MAPK/ERK signaling pathway, and promoting tumor growth and GC cell migration and invasion (54).

**Figure 2:**
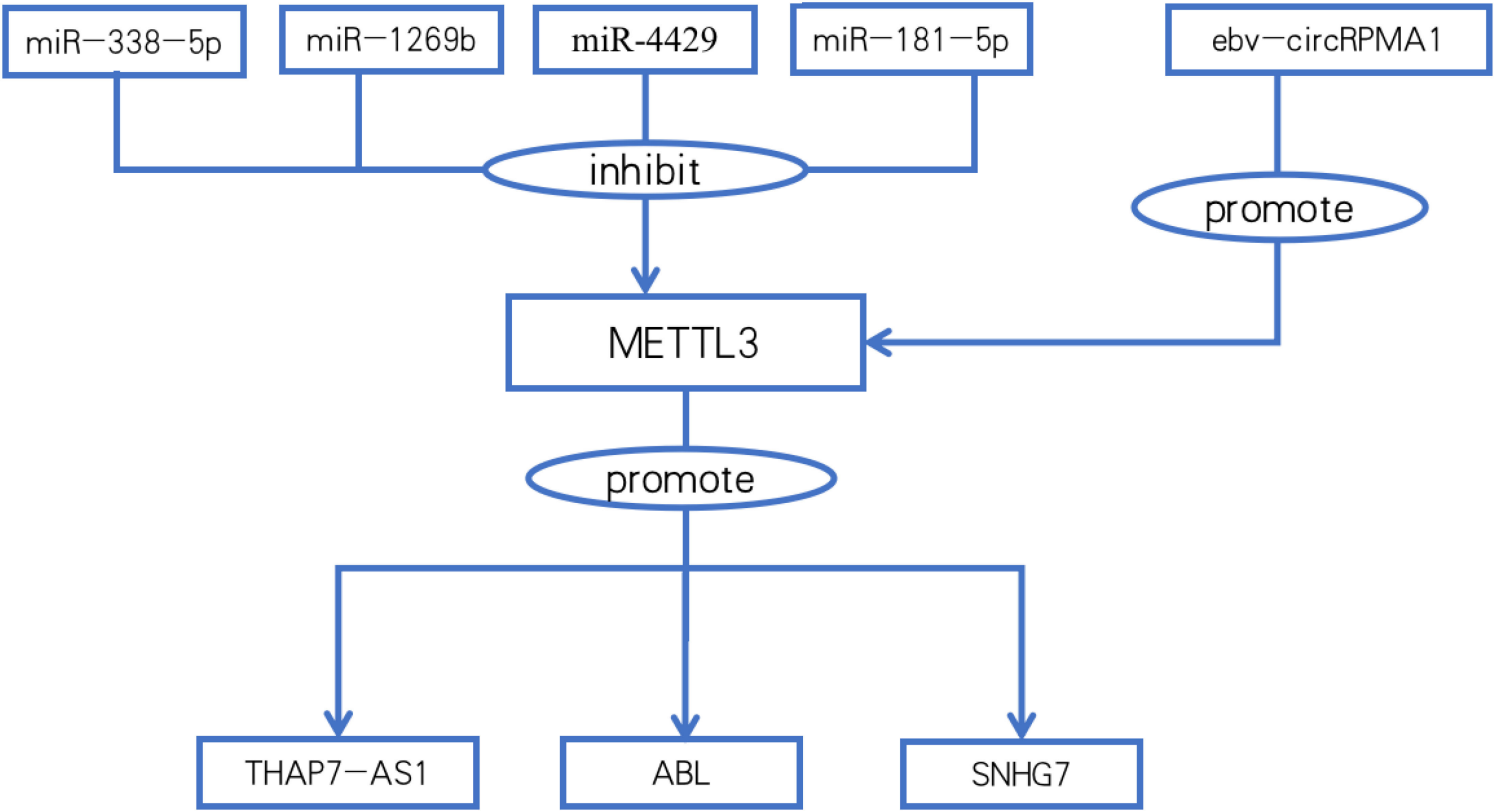
Relationships between METTL3 and noncoding RNAs.

Autophagy is essential for maintaining optimal cellular function during energy metabolism and helps inhibit the progression of various diseases, such as aging and cancer. Autophagy also degrades cancer-promoting proteins. Dysregulation of autophagy can lead to a variety of pathological processes, such as cancer chemotherapy resistance. ARHGAP5-AS1 enhances the interaction between ARHGAP5 mRNA and HuR (reader) by recruiting METTL3, maintaining ARHGAP5 mRNA stability, and promoting its expression. High ARHGAP5 expression is associated with chemoresistance and shorter overall and progression-free survival in GC cells (55). Similarly, DNA modification is DNA methylation catalyzed by DNA methyltransferase 3a (DNMT3a). Linc00942 recruits METTL3 to DNMT3a mRNA and stabilizes DNMT3a mRNA through “IGF2BP3/HuR-dependent” m6A modification, resulting in an increase in the level of DNA modification and chemotherapy resistance. Linc00942 autophagy, which is mediated by p62, restores gastric cancer cell sensitivity to chemotherapeutic agents by decreasing DNA methylation levels (56). (**Table 4**; **Figure 2**). These results suggest that in addition to inducing lncRNA autophagy, DNA methylation-targeted therapy provides a new way to solve the problem of chemoresistance (**Figure 2**).

**Table 4:** Overview of the relationship between METTL3 and noncoding RNAs.

Upregulator	Target	Mechanism	Biological function	Reference
miR-1269b	METTL3	Inhibit the expression of METTL3	Restrain the proliferation, migration and invasion of GC cells	(43)
miR-4429	METTL3	Inhibit the expression of METTL3	Suppress the proliferation and enhance apoptosis	(44)
miR-181-5p	KLHL5	Reduce m6A level and METTL3 expression by negatively regulates KLHL5 expression	Inhibit the proliferation, migration, invasion and lung metastasis of GC	(45)
miR-338-5p	METTL3	Inhibit the expression of METTL3	Suppress the proliferation and migration of GC	(46)
ebv-circRPMA1	METTL3	Promote the transcription of METTL3	Promote the progression and lung metastasis of EBV-associated GC	(47, 48)
METTL3	lncRNA THAP7-AS1	Enhance the stability of THAP7-AS1 transcription	Promote GC cell growth, migration and invasion	(51)
METTL3	lncRNA ABL	Maintain the stability of ABL	Inhibit apoptosis of GC cells	(52)
METTL3	lncRNA SNHG7	Upregulate the expression of SNHG7	Lead to the malignant transformation of MNNG exposure cells and promote GC cells migration and invasion	(53)
LINC02253	METTL3	Recruit METTL3 to KRT18 mRNA	Promote the migration and invasion of GC cells	(54)
ARHGAP5-AS1	METTL3	Recruit METTL3 to ARHGAP DNA/mRNA	Promote chemoresistance of GC	(55)
LINC00942	METTL3	Recruit METTL3 to DNA methyltransferase 3a mRNA	Promote chemoresistance of GC	(56)

**Table 4** circRNA, circular RNA; EBV, Epstein–Barr virus; LncRNA, long noncoding RNA; miRNA, microRNA-; GC, gastric cancer; METTL3, methyltransferase-like 3.

### METTL3 functions independent of enzyme activity

The subcellular localization of METTL3 demonstrated that cytoplasmic METTL3 regulates translation initiation by interacting with cytoplasmic METTL3-related proteins, which in turn promotes gastric carcinogenesis independent of m6A modification. The translation initiation factor PABPC1 binds to the 3 poly(A) tail. PABPC1 interacts with eIF4F to form a closed-loop conformation of RNA, in which METTL3 contributes to PABPC1 translation initiation by directly binding to PABPC1 to promote the interaction between PABPC1 and eIF4F to maintain the loop RNA structure and regulate translation. The METTL3-PABPC1 complex promotes the translation of histone modification genes to deactivate histones to regulate epigenetic inheritance in GC cells. METTL3-PABPC1 does not promote methylation (57). Studies have shown that acetylation of METTL3 is associated with the progression of gastric cancer. Acetylated METTL3 cannot effectively promote RNA translation. The acetyl group blocks the binding of METTL3 to EIF3H and destroys the ring structure. Acetylation of METTL3 occurs on amino acids related to mRNA translation and has nothing to do with the methyltransferase activity of METTL3 (58). Ianniello Z et al. demonstrated that in cells from patients with chronic myeloid leukemia, METTL3 in the nucleus is only responsible for transferring methyl groups to PES1 mRNA, while the enhancement of PES1 protein synthesis is the result of the action of METTL3 in the cytoplasm (59).

## Discussion

The occurrence and progression of GC are closely related to high METTL3 expression levels, as shown in recent studies. METTL3 oncogene mRNA expression was significantly higher in GC tissues than in normal tissues. Tumor suppression can be achieved by downregulating METTL3 expression. Consequently, a novel strategy for the treatment of GC is to target METTL3. YANKOVA et al. identified a compound called STM2457 that binds directly to the SAM binding pocket. It inhibits the catalytic activity of METTL3 by competing with SAM but does not destroy the enzyme structure (60). In addition, some natural substances, such as quercetin and coptisine chloride, are antitumor small-molecule drugs. These compounds inhibited the enzymatic activity of METTL3. However, the mechanism by which these natural substances bind to METTL3 remains unclear (61). Manna et al. discovered several natural products that can bind SAM-binding pockets via methods such as docking and molecular dynamic simulation. These natural products have stronger binding properties than STM2457 does (62). These results suggest that the role of METTL3 small-molecule drugs in tumor treatment involves competing with SAM as well as disrupting the methyltransferase activity of METTL3. However, methylation is not the only mechanism by which METTL3 promotes gastric cancer progression. It has been demonstrated that KH12, formed by PROTAC technology, degrades METTL3 via the ubiquitin−proteasome pathway (63). WD6305 is a newly developed PROTAC degrader that degrades METTL3 by ubiquitination. METTL14, as a protein that interacts with METTL3, is also degraded at the same time (64). These discoveries provide a new theoretical basis for targeted therapy of GC unrelated to the m6A methylation function of METTL3.

METTL3 leads to the upregulation of oncogenes or the downregulation of tumor suppressors, which affects the malignant progression of GC. However, the molecular mechanisms regulating gastric carcinogenesis and progression are complex, and existing findings are just the tip of the iceberg. Notably, METTL3 is the only methyltransferase with enzymatic activity among m6A regulators, such as methyltransferases (writers), demethylases (erasers), and m6A recognition proteins (readers) (65). Stable knockdown of the demethylase FTO can lead to upregulation of E-calmodulin and downregulation of waveform proteins in a variety of GC cell lines, suggesting that FTO may participate in generating stable and overexpressed EMT-related genes, thereby exerting carcinogenic effects (66). FTO can demethylate HOXB13 mRNA, resulting in high HOXB13 expression. This is a deterioration in the condition of patients with stomach cancer. HOXB13 promotes IGF-1R transcription and activates the PI3K/AKT/mTOR pathway. In contrast, when FTO levels are reduced, the malignant development of cancer cells is inhibited (67). Therefore, METTL3 is not the only factor that determines m6A levels, and the factors and mechanisms that regulate m6A levels need to be further investigated in more comprehensive experiments.

METTL3 mostly causes GC progression and drug resistance in gastric cancer. Targeting METTL3 can improve the efficacy of immunotherapy, alleviate chemotherapy resistance, and inhibit tumor growth, which is an advantage of using METTL3 as a target for gastric cancer. However, their disadvantages cannot be ignored. According to Zhang et al., METTL3 may act as a cancer suppressor in GC cell lines and other tumors. Low m6A signaling is strongly associated with the malignant progression of GC. Decreased m6A levels mediate the activation of signals such as Wnt and PI3K-Ak, whereas increased m6A levels mediate the activation of tumor suppressor signals (68). Sun et al. reported that METTL3 promoted the maturation of pri-miR-17-92 and that miR-17-92 activated the AKT/mTOR pathway and improved the sensitivity of GC to chemotherapy (69).

In addition to targeting METTL3 for the treatment of GC, many upstream regulators of METTL3 can also be targeted. For example, H3K27ac binds to the METTL3 promoter to regulate METTL3 expression, and HOXA10 activates the expression of TGFB2 through transcriptional regulation. Increased Smad2/3 expression results from activation of the TGF-β-Smad2/3 pathway. Smad2/3 bind directly to the METTL3 promoter, participate in regulating METTL3 transcription, increase the m6A levels of Slug and Snail in GC cells, accelerate the translation of Snail and Slug, and induce EMT progression (29). In addition, molecules such as Linc00942 and ARHGAP5-AS1 can promote METTL3 recruitment, and this effect can be attenuated by inducing autophagy. In addition, in the immunotherapy of tumors, the methylation of core molecules regulated by METTL3 determines the TMB of different GC subtypes. In cells with high METTL3 expression, the level of PD-L1 expression is also greater, and patients with a high TMB have greater sensitivity to immune checkpoint inhibitors, such as paclitaxel. Some retrospective studies have demonstrated that METTL3 in GC is the main cause of poor overall survival and is correlated with advanced PT, PN, and TNM stages; a tumor size greater than 5 cm; and vascular infiltration (70, 71). Therefore, METTL3 and METTL3-related molecules can be used as clinical targets for patients with GC and as predictive and prognostic biomarkers for GC.
